# A Novel platform for precision quantitative analyses of neuropathology in Alzheimer’s disease

**DOI:** 10.1002/alz.087480

**Published:** 2025-01-03

**Authors:** C Dirk Keene

**Affiliations:** ^1^ University of Washington, Seattle, WA USA

## Abstract

**Background:**

The BRAIN Initiative has stimulated development of novel single cell and spatial molecular approaches to understand human brain structure and function. However, traditional methods for human brain specimen collection, including retrospective archival tissues, have not been optimized for these latest methods. A modernized approach that optimizes tissue quality, anatomical precision, and comprehensive, quantitative neuropathological assessments is needed to maximize the impact of the tremendous investment and remarkable technological advances in human neuroscience research.

**Method:**

We developed a modernized methodology that incorporates rapid neuroimaging, hemibrain embedding in dental alginate, consistently thin (4mm) coronal slicing, and alternating fixation or freezing in supercooled isopentane. Fixed tissues are comprehensively sampled using functionally‐informed, network‐based approach. Traditional neuropathological examination is combined with quantitative neuropathological analysis of digital images of pathologic peptides and markers of neurons, glia, and neuroinflammation. We developed a temporally‐based case series (all donor brains collected during a certain time frame, excluding frontotemporal lobar degeneration) from participants in the UW ADRC Clinical Core and the Kaiser Adult Changes in Thought (ACT) study. Brain regions for examination were chosen to model the temporal progression of AD neuropathology; samples for molecular studies we from adjacent frozen slices.

**Result:**

Eighty‐four brains were included in this study from donors (∼60% female) ranging from no cognitive impairment to dementia. Tissue quality metrics (RIN, pH, PMI) were high, nuclear yields for single cell omics were robust (with rare exceptions), and spatial transcriptomics performed well, with minimal tissue freezing artifact. Traditional neuropathological examination confirmed a spectrum of not to high AD neuropathologic change (ADNC), with a subset of the cohort exhibiting some level of one or more age‐related comorbid pathologies. HALO‐based analysis of digital pathology of beta amyloid, pTau, pTDP‐43, alpha‐synuclein, NeuN, GFAP, and Iba‐1 across cortical layers resulted in an array of quantitative variables which were used to generate continuous pseudo progression scores for the cohort that informed molecular omics analyses.

**Conclusion:**

Development and implementation of improved methods for tissue collection, characterization, and preservation, combined with enhanced sampling and integrated neuropathology, resulted in a robust tissue and data resource to support modern molecular cell‐types and pathway‐based analyses.

